# Real-Time Coseismic Displacement Retrieval Based on Temporal Point Positioning with IGS RTS Correction Products

**DOI:** 10.3390/s21020334

**Published:** 2021-01-06

**Authors:** Yuanfan Zhang, Zhixi Nie, Zhenjie Wang, Huisheng Wu, Xiaofei Xu

**Affiliations:** College of Oceanography and Space Informatics, China University of Petroleum, Qingdao 266580, China; z18010038@s.upc.edu.cn (Y.Z.); sdwzj@upc.edu.cn (Z.W.); wuhuisheng@upc.edu.cn (H.W.); xuxiaofei.qd@gmail.com (X.X.)

**Keywords:** IGS RTS, high-rate GNSS, coseismic displacement, TPP

## Abstract

With the rapid development of the global navigation satellite system (GNSS), high-rate GNSS has been widely used for high-precision GNSS coseismic displacement retrieval. In recent decades, relative positioning (RP) and precise point positioning (PPP) are mainly adopted to retrieve coseismic displacements. However, RP can only obtain relative coseismic displacements with respect to a reference station, which might be subject to quaking during a large seismic event. While PPP needs a long (re)convergence period of tens of minutes. There is no convergence time needed in the variometric approach for displacements analysis standalone engine (VADASE) but the derived displacements are accompanied by a drift. Temporal point positioning (TPP) method adopts temporal-differenced ionosphere-free phase measurements between a reference epoch and the current epoch, and there is almost no drift in the displacement derived from TPP method. Nevertheless, the precise orbit and clock products should be applied in the TPP method. The studies in recent years are almost based on the postprocessing precise orbits and clocks or simulated real-time products. Since 2013, international GNSS service (IGS) has been providing an open-access real-time service (RTS), which consists of orbit, clock and other corrections. In this contribution, we evaluated the performance of real-time coseismic displacement retrieval based on TPP method with IGS RTS correction products. At first, the real-time precise orbit and clock offsets are derived from the RTS correction products. Then, the temporal-differenced ionosphere-free (IF) combinations are formed and adopted as the TPP measurements. By applying real-time precise orbit and clock offsets, the coseismic displacement can be real-timely retrieved based on TPP measurements. To evaluate the accuracy, two experiments including a stationary experiment and an application to an earthquake event were carried out. The former gives an accuracy of 1.8 cm in the horizontal direction and 4.1 cm in the vertical direction during the whole period of 15-min. The latter gives an accuracy of 1.2 cm and 2.4 cm in the horizontal and vertical components, respectively.

## 1. Introduction

With the rapid development of the global navigation satellite system (GNSS), high-rate GNSS has been widely used for seismology in the past two decades [1,2,3]. Based on retrieved high-precision GNSS coseismic displacements, earthquake magnitude and finite fault slip can be accurately estimated, and they can be further used for rapid hazard assessment and earthquake early warning (EEW) [4,5,6,7,8,9].

Relative positioning (RP) and precise point positioning (PPP) are mainly adopted to retrieve coseismic displacements [10]. RP technique is able to achieve 1–2 cm positioning accuracy and it is widely applied to record strong ground motion for further centroid moment tensor determination [11], fault model estimation [12] and early warning [13,14,15]. However, it only derives relative coseismic displacements with respect to a reference station, which might be subject to quaking during a large seismic event. PPP technique provides absolute coseismic displacements under a global reference frame without requiring a local GNSS reference station [16,17,18,19]. Nevertheless, it has limited accuracy because of unresolved integer-cycle ambiguities [20]. In recent years, precise point positioning with ambiguity resolution (PPP-AR) has been developed to improve the positioning accuracy of PPP method [21,22,23]. It can provide comparable accuracy as that of RP technique by applying precise orbit, clock, uncalibrated phase delay (UPD) or fractional cycle bias (FCB) products [24,25]. However, the limitation of PPP-AR is that a long (re)convergence period of tens of minutes is needed. The accuracy of the PPP-derived/PPP-AR-derived coseismic displacement might be decreased when an earthquake happens by coincidence during the PPP/PPP-AR (re)convergence period [26].

In 2011, Colosimo et al. proposed variometric approach for displacement analysis standalone engine (VADASE) [27]. Based on epoch-differenced carrier phase observations and broadcast ephemeris, the changes of positions are estimated by employing least-square (LS) estimation in the VADASE method [28,29]. Coseismic displacements are obtained by a single integration of the changes of positions. Compared with PPP technique, there is no convergence time needed in the VADASE method but the derived displacements are accompanied by a drift due to potential uncompensated errors [30,31]. Branzanti et al. assumed that the drift could be effective eliminated within a few minutes by using a linear trend removal [32]. Hung et al. applied modified sidereal filtering and spatial filtering to decrease the drift trend [33,34]. However, these existing detrending methods need to use postprocessed preseismic and coseismic displacements to calculate linear and nonlinear trend terms. Therefore, they cannot meet the demand of real-time coseismic displacement retrieval.

In order to remove the drift in the displacement obtained by VADASE method, Li et al. and Guo et al. presented a temporal point positioning (TPP) method [35,36]. Instead of differencing carrier phase measurements between adjacent epochs in the VADASE method, TPP method adopts temporal-differenced measurements between a reference epoch and the current epoch, and there is almost no drift in the displacement derived from TPP method [37]. Chen et al. retrieved the coseismic displacements of the Illapel Mw 8.3 earthquake and the Manila Trench Mw 8.0 earthquake with TPP method and found the accuracy of retrieved coseismic displacements with GPS/GLONASS and GPS/BDS observations was significantly better than that derived with GPS-only measurements [38,39]. Nevertheless, the precise orbit and clock products should be adopted in the TPP method. The studies above are almost based on the postprocessing precise orbits and clocks or simulated real-time products.

To meet the growing demands of real-time precise applications, international GNSS service (IGS) has been providing an open-access real-time service (RTS) since 2013, which consists of orbit, clock and other corrections. The RTS correction products are formatted into state space representation (SSR) messages according to the standard of Radio Technical Commission for Maritime Services (RTCM) [40]. It is transmitted over the internet based on the Networked Transport of RTCM via Internet Protocol (NTRIP) [41]. In spite of numerous studies to evaluate the performance of real-time PPP with RTS correction products [42,43,44,45,46], there are few studies, to our knowledge, devoted to real-time coseismic displacement retrieval based on TPP method with RTS correction products. 

In this contribution, we evaluated the performance of real-time coseismic displacement retrieval based on TPP method with RTS correction products. At first, we derived the real-time precise orbit and clock offsets from the RTS correction products. Then, the temporal-differenced ionosphere-free (IF) combinations are formed and adopted as the TPP measurements. By applying real-time precise orbit and clock offsets, the coseismic displacement can be real-timely retrieved based on TPP measurements. To evaluate the performance of coseismic displacement derived from TPP method based on IGS RTS correction products, the 1 Hz GPS data obtained from 33 IGS stations were collected and the displacements were obtained based on TPP method with Centre National d’Etudes Spatiales (CNES) real-time correction products. The accuracies of obtained displacements were assessed. As comparison, we also calculated two displacement results based on TPP method with final products obtained from the Center of Orbit Determination in Europe (CODE) and VADASE method with broadcast ephemeris. In addition, an application to capture coseismic waveform of 2016 Mw 7.8 Kaikōura earthquake was further conducted. The accuracies of the real-time retrieved coseismic displacements were validated with the displacements derived from postprocessed PPP method as references.

The rest of the paper is organized as follows. In Section 2, the recovery of precise orbit and clock offset with RTS corrections is introduced. The coseismic displacement retrieval method is discussed in detail. In Section 3, the performance is evaluated with high-rate GNSS data collected from stationary stations and real Kaikōura earthquake event. Conclusions are summarized in the last section.

## 2. Methods

### 2.1. Recovery of Precise Orbit and Clock Offset with IGS RTS Correction Products

As mentioned above, IGS RTS corrections are formatted into SSR messages. The SSR orbit correction message contains the parameters for orbit corrections in the radial, along-track and cross-track directions $\delta O_{r},\;\delta O_{a},\;\delta O_{c}\;$ and their velocities $\delta {\dot{O}}_{r},\;\delta {\dot{O}}_{a},\;\delta {\dot{O}}_{c}$ at the SR epoch time. The orbit corrections in the radial, along-track and cross-track directions can be calculated as follows [40]

(1) $$\begin{array}{c} \delta O=\left[\begin{array}{c} \delta O_{r}\\ \delta O_{a}\\ \delta O_{c} \end{array}\right]+\left[\begin{array}{c} \delta {\dot{O}}_{r}\\ \delta {\dot{O}}_{a}\\ \delta {\dot{O}}_{c} \end{array}\right]\left(t-t_{0}\right) \end{array}$$

where t and $t_{0}$ are the current and reference time, and the reference time is computed from the SSR epoch time plus half of the SSR update interval. 

**Figure 1:**
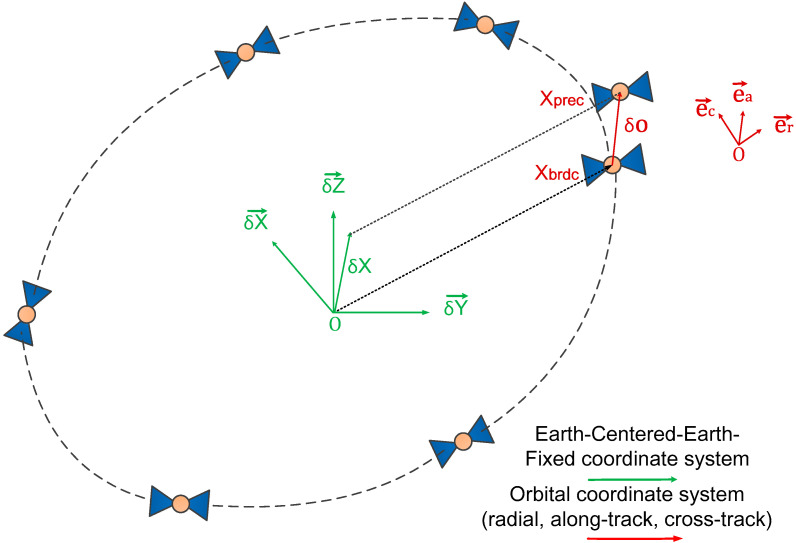
The geometry sketch of real-time precise orbits recovered from IGS RTS correction products.

As shown in Figure 1, the corrected precise satellite position $X^{s}{}_{prec}\left(t\right)$ in the Earth-center-Earth-fixed (ECEF) frame can be expressed as [40,47]

(2) $$\begin{array}{c} X^{s}{}_{prec}\left(t\right)=X^{s}{}_{brdc}\left(t\right)+\left[e_{r},e_{a},e_{c}\right]\delta O \end{array}$$

and

(3) $$\begin{array}{c} \left\{\begin{array}{c} e_{a}=\frac{{\dot{X}}^{s}{}_{brdc}\left(t\right)}{\left|{\dot{X}}^{s}{}_{brdc}\left(t\right)\right|}\\ e_{c}=\frac{X^{s}{}_{brdc}\left(t\right)\times {\dot{X}}^{s}{}_{brdc}\left(t\right)}{\left|X^{s}{}_{brdc}\left(t\right)\times {\dot{X}}^{s}{}_{brdc}\left(t\right)\right|}\\ e_{r}=e_{a}\times e_{c} \end{array}\right.\;\;\;\;\; \end{array}$$

where $X^{s}{}_{brdc}\left(t\right)$ and ${\dot{X}}^{s}{}_{brdc}\left(t\right)$ are satellite position and velocity computed with the broadcast ephemeris.

SSR clock correction message is streamed in the form of polynomial coefficients $a_{0}$, $a_{1}$ and $a_{2}$. The clock correction at the time of t can be expressed as [40]

(4) $$\begin{array}{c} \delta C=a_{0}+a_{1}\left(t-t_{0}\right)+a_{2}{\left(t-t_{0}\right)}^{2} \end{array}$$

The corrected precise satellite clock offset $dt^{s}{}_{prec}\left(t\right)$ can be computed as [40,48]

(5) $$\begin{array}{c} dt^{s}{}_{prec}\left(t\right)=dt^{s}{}_{brdc}\left(t\right)+\frac{\delta C}{c} \end{array}$$

where $dt^{s}{}_{brdc}\left(t\right)$ is the satellite clock offset at the time of t computed with the broadcast ephemeris, c represent the speed of light.

### 2.2. Real-Time Coseismic Displacement Retrieval Method Based on Real-Time Precise Orbits and Clock Offsets

The GNSS raw phase measurement reads as [49,50]

(6) $$\begin{array}{c} L_{j}=\rho +c\cdot \delta t_{r}-c\cdot \delta t^{s}+T-\kappa_{j}\cdot I+\lambda_{j}\cdot N_{j}+b_{r,j}-b_{j}^{s}+\varepsilon_{L_{j}} \end{array}$$

where the subscript j represents the carrier frequency number; ρ is the geometric distance between the satellite and receiver; c denotes the speed of light; $\delta t_{r}$ and $\delta t^{s}$ are clock offsets at the receiver- and satellite-end; T denotes tropospheric delay along the path; I denotes the ionospheric delay for the first carrier frequency and $\kappa_{j}=f_{1}^{2}/f_{j}^{2}$ is the ionospheric scalar factor for jth carrier frequency with a frequency value of $f_{j}$; $\lambda_{j}$ is the wavelength and $N_{j}$ is the integer ambiguity; $b_{r,j}$ and $b_{j}^{s}$ represent receiver- and satellite-dependent uncalibrated phase delays; $\varepsilon_{L_{j}}$ is the measurement noise including thermal and multipath. Although not mentioned in the GNSS raw phase measurement, the other corrections including Sagnac effect, satellite/receiver antenna phase center offset (PCO) and phase center variation (PCV) [51], special relativistic effect and Shapiro time delay [52], phase windup effect [53] and site displacements causing by the influence of solid tide, ocean loading and pole tide [54], are defaulted to be precisely corrected by applying corresponding models.

The IF phase combination measurement equation is expressed as [10,50]

(7) $$\begin{array}{c} L_{IF}=a\cdot L_{1}+\left(1-\alpha \right)\cdot L_{2}=\rho +c\cdot \delta t_{r}-c\cdot \delta t^{s}+\lambda_{IF}\cdot N_{IF}+T+\varepsilon_{L_{IF}} \end{array}$$

where $\alpha =f_{1}^{2}/\left(f_{1}^{2}-f_{2}^{2}\right)$; $\lambda_{IF}\cdot N_{IF}=\alpha \cdot \left(b_{r,1}-b_{1}^{s}+\lambda_{1}N_{1}\right)+\left(1-\alpha \right)\cdot \left(b_{r,2}-b_{2}^{s}+\lambda_{2}N_{2}\right)$ denotes ionosphere-free ambiguity; $\varepsilon_{IF}=\alpha \cdot \varepsilon_{1}+\left(1-\alpha \right)\cdot \varepsilon_{2}$ is measurement noise of the ionosphere-free phase combination.

**Figure 2:**
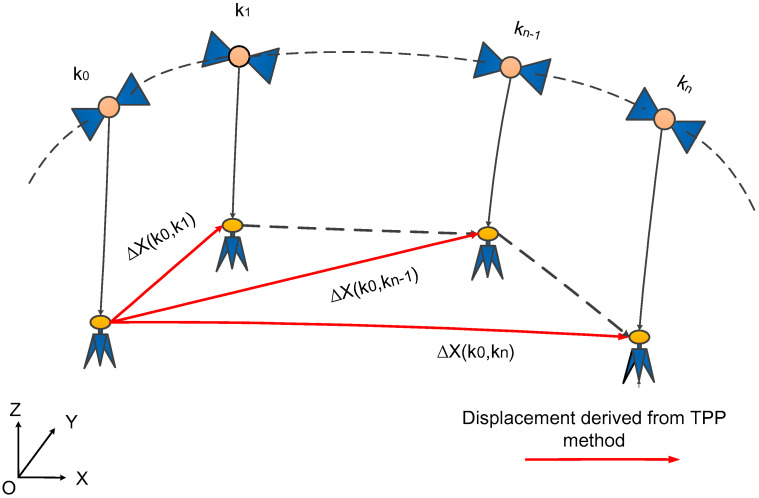
The sketch of real-time displacement retrieval method with real-time precise orbits and clocks.

TPP method obtains the displacement of a single receiver by employing the temporal-differenced IF measurements [35,36,37,38], as depicted in Figure 2. If the GNSS raw observations are continuous, the real-valued ambiguities $N_{IF}$ can be deemed as constants, which is eliminated through the temporal-differenced operation [35,37]. Meanwhile, if the meteorological condition does not change abruptly in a few minutes, the residual part of T is limited to centimeter-level after being corrected with a priori tropospheric delay model [55]. As a result, the temporal-differenced IF measurement equation can be expressed as follows

(8) $$\begin{array}{c} \Delta L_{IF}=L_{IF}\left(k_{i}\right)-L_{IF}\left(k_{0}\right)=\Delta \rho +c\cdot \Delta \delta t_{r}+\Delta \varepsilon_{L_{IF}} \end{array}$$

where $k_{i}$ represents the $i\mathrm{th}\;\left(i=0,1,\cdots ,n\right)$ sampling epoch of GNSS raw observations; Δ denotes the difference operator between the epoch $k_{0}$ and the epoch $k_{i}$; $\Delta L_{IF}$ represents the temporal-differenced IF measurement; Δρ denotes the temporal-differenced geometric distance between the satellite and receiver; $\Delta \delta t_{r}$ stands for the temporal differenced receiver clock offset; $\Delta \varepsilon_{L_{IF}}$ represents the temporal-differenced IF measurement noise.

After applying the real-time precise orbits and clock offsets derived from RTS correction products, the temporal-differenced IF measurement equation can be linearized as follows

(9) $$\begin{array}{c} \Delta l_{IF}=-e\cdot \Delta X-\Delta e\cdot X\left(k_{0}\right)+c\cdot \Delta \delta t_{r}+\Delta \varepsilon_{L_{IF}} \end{array}$$

where $\Delta l_{IF}$ denotes the observed-minus-computed temporal-differenced IF measurement residuals; e denotes the unit vector of the direction from receiver to satellite at the current epoch $k_{i}$ and ΔX presents the position increment with respect to the reference epoch; Δe stands for the change of the line-of-sight vector and $X\left(k_{0}\right)$ is the position at the reference epoch, which can be obtained through routinely postprocessing RP or PPP day by day. The unknowns only include the position increment ΔX and the receiver clock bias of $\Delta \delta t_{r}$ and they can be estimated by least-square (LS) method.

**Figure 3:**
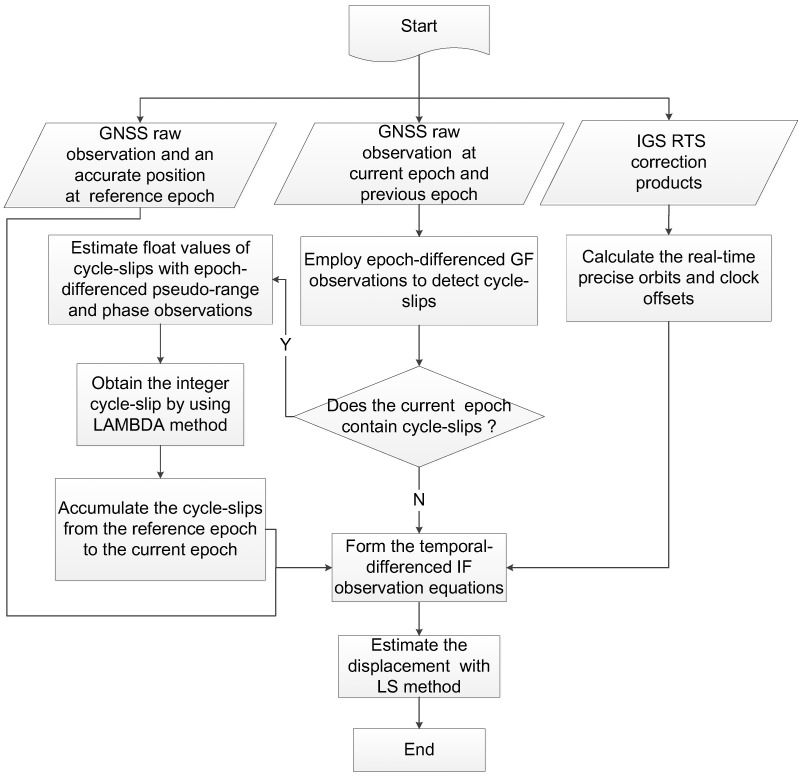
Flowchart of the real-time displacement retrieval with TPP method based on IGS RTS correction products.

The whole procedure of TPP method based on RTS correction products is displayed in Figure 3. At first, we employ epoch-differenced geometry free (GF) combinations to detect cycle-slips. Once there are cycle-slips detected, epoch-differenced pseudorange and phase observations are used to estimate a float solution of the cycle-slips, and then the LAMBDA method is further adopted to obtain an integer solution [56,57]. The integer cycle-slips are accumulated from the reference epoch to the current epoch. The temporal-differenced IF measurement is corrected with the accumulated integer cycle-slip values. At the same time, the precise orbits and clock offsets computed from RTS correction products and a precise position of reference epoch are applied to linearize the temporal-differenced IF observation equation. Finally, the coseismic displacement can be estimated with the LS method.

## 3. Experiments and Results

To evaluate the performance of TPP method with RTS correction products, two experiments including a stationary experiment and an application to an earthquake event were carried out. During the time period of the stationary experiment and earthquake event, the CNES CLK93 real-time stream was received from BKG NTRIP Client (BNC) software and stored in a file. Both experiments were simulated by processing the collected data in the postprocessed mode. As comparison, the displacements were also retrieved with TPP method based on 15-min precise orbit products and 5-s precise clock products from CODE, and VADASE method based on broadcast (BRDC) ephemeris. The three processing schemes are presented in Table 1. For the sake of convenience, these three processing schemes are sequentially denoted as TPP+RTS, TPP+CODE and VADASE+BRDC in the following.

The software for the real-time coseismic displacement retrieval were programmed by using the C language following the method of TPP and VADASE. During the displacement estimation at each epoch, the computational time can be limited to several milliseconds. Only GPS L1/L2 observations are employed to estimate displacements both in stationary and seismic application. The cut-off elevation angle was set to 10 degrees. The accurate position at the reference epoch was calculated by Natural Resources Canada online Precise Point Positioning (CSRS-PPP) tool by using three-hour observations before the reference time (https://webapp.geod.nrcan.gc.ca/). Table 2 summarizes the data processing strategies for TPP method in detail.

### 3.1. Stationary Experiment with Global IGS Stations

**Figure 4:**
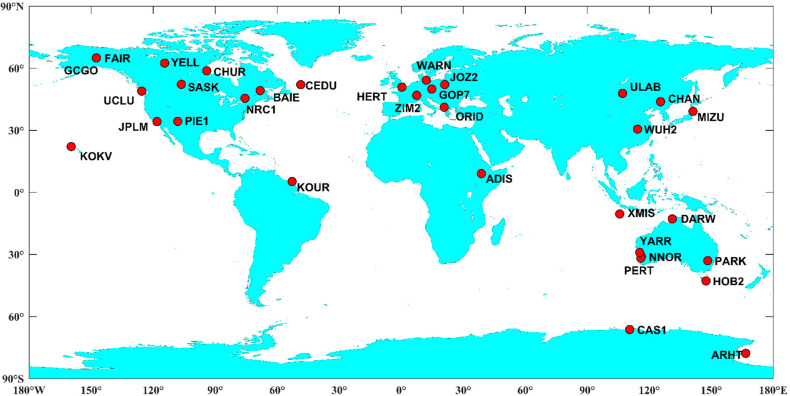
Distribution of the collected 33 IGS stations.

To assess the performance of TPP method with RTS correction products, 33 globally distributed IGS stations were selected. The distribution of the stations is shown in Figure 4. The observations from 05:45:00 to 05:59:59 on 1 January 2020 in GPS time were collected and processed. The time period of 15 min is significantly longer than the duration of typical earthquake, which is generally last for less than a few minutes. The static experiment gives us an overall impression about the accuracy of the displacements derived from TPP method with real-time orbit and clock products. As mentioned above, the displacements based on TPP method with CODE final products and VADASE method with broadcast ephemeris were also obtained for comparison. Considering that the selected IGS stations are stationary, the displacement should be zero at each epoch, which can be used as references. All displacements derived from different schemes were compared with the references to validate the accuracy. In order to evaluate the performance of real-time coseismic displacement retrieval, no linear or nonlinear detrending procession such as Shu et al. and Hung et al. [30,34] was applied to the displacements derived from VADASE method.

**Figure 5:**
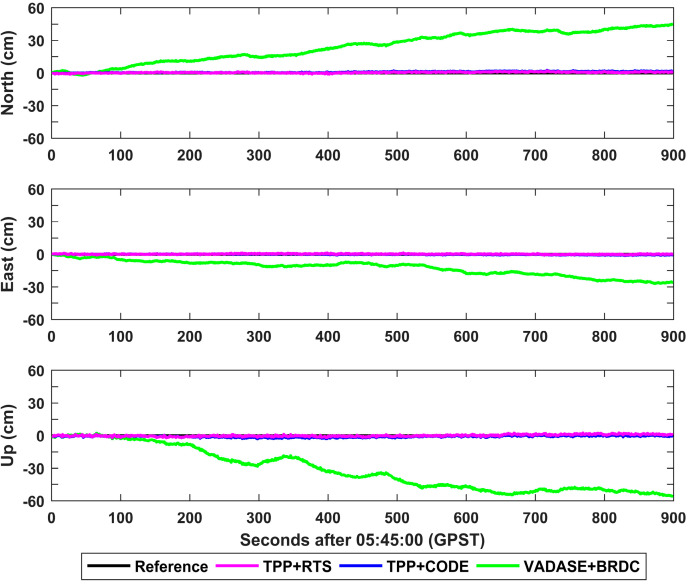
The retrieved displacements in north, east and up components for different schemes at station MIZU.

The displacement time series at a typical station MIZU is shown in Figure 5. The average values of displacements in north, east and up directions are 2.0 cm, 1.3 cm and 2.5 cm for TPP+RTS. Regarding TPP+CODE, the average values of the retrieved displacements in north, east and up directions are 2.5 cm, 1.1 cm and 3.2 cm, respectively. However, there is an evident drift in the displacements derived from VADASE+BRDC. The displacements in north, east and up directions reach up to 45.0 cm, 27.0 cm and 56.1 cm at the end of time series. 

**Figure 6:**
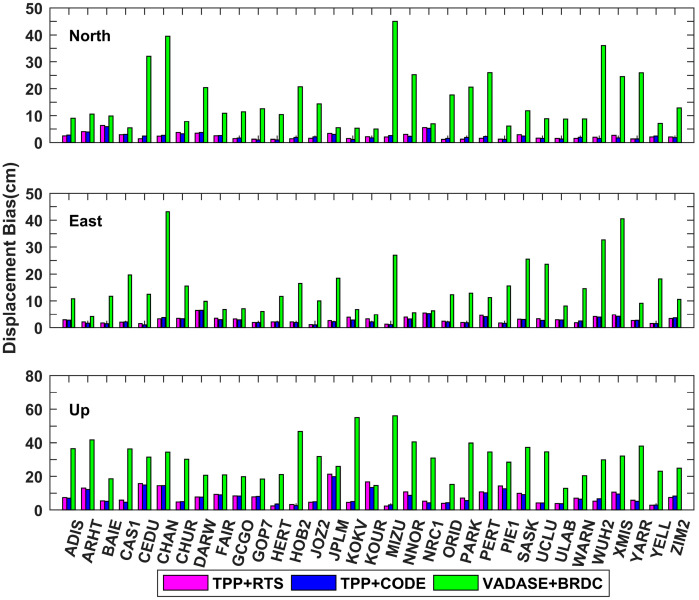
The average values of the derived displacements in north, east and up directions for different schemes at each station.

**Figure 7:**
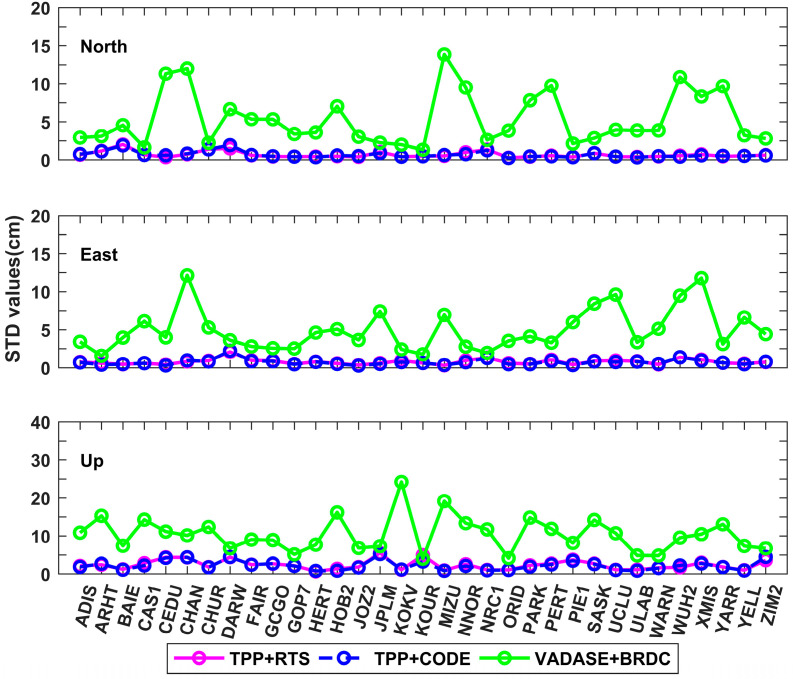
The standard deviation (STD) values of the derived displacements in north, east and up directions for different schemes at each station.

The average and standard deviation (STD) values of the derived displacements during the whole time period were calculated for 33 IGS stations, and they are shown in Figure 6 and Figure 7. The mean values of average displacements in north, east and up directions are 2.3 cm, 2.9 cm and 8.1 cm for TPP+RTS, which are at the same level as those of TPP+CODE. However, the mean values of average displacements in north, east and up directions are 15.9 cm, 14.8 cm and 30.4 cm for VADASE+BRDC. The means of STD values in north, east and up directions are 0.7 cm, 0.8 cm and 2.4 cm for TPP+RTS, and similarly they are in close proximity to those of TPP+CODE. The means of STD values of VADASE+BRDC in north, east and up directions are 5.1 cm, 4.9 cm and 10.4 cm, which are significantly larger than the results of TPP+RTS.

The average root mean square (RMS) values of the retrieved displacements over 33 IGS stations for three different schemes are summarized in Table 3. The average RMS values are 1.8 cm and 4.1 cm in horizontal and vertical directions for TPP+RTS, which are at the same level as that of TPP+CODE. While the average RMS values of VADASE+BRDC reach up to 12.1 cm and 15.7 cm in horizontal and vertical directions, respectively. The displacements derived from TPP+RTS are highly consistent with the displacements derived from TPP+CODE. In a word, TPP method with real-time orbit and clock products and CODE final products show nearly equivalent performance of displacement retrieval. Significant improvement is shown in the accuracy of retrieved real-time displacement compared to that of VADASE method.

### 3.2. Application to Earthquake Monitoring: The 2016 Mw 7.8 Kaikōura Earthquake

**Figure 8:**
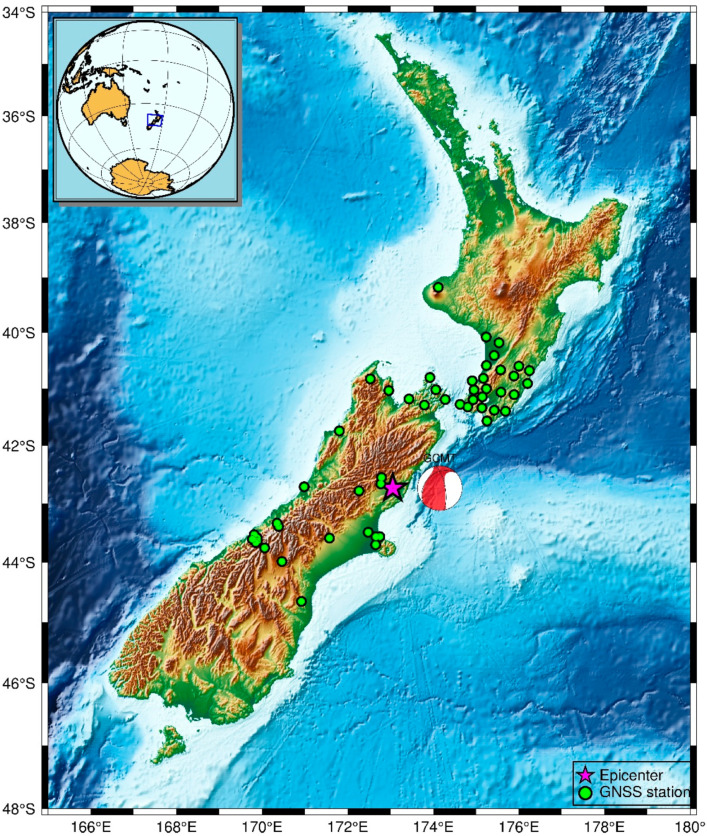
Location of the 2016 Kaikōura earthquake epicenter and the distribution of the selected 1 Hz GPS stations.

The 2016 Mw 7.8 Kaikōura earthquake happened in the South Island of New Zealand at 11:02:56 (UTC) on 13 November. The hypocentral was at a relatively shallow depth of 15.1 km and its epicenter was located at 42.737° S, 173.054° E (https://earthquake.usgs.gov/earthquakes/). The earthquake rupture caused a tsunami which was up to 3 m at Kaikōura [58]. The impacts of the Kaikōura earthquake were enormous. Thousands of people were affected with significant damage to transportation networks and other infrastructure as well as disruption to the agriculture and tourism industries [59]. This seismic event was successfully recorded by a great deal of GNSS stations. In this experiment, high-rate GPS observations (1 Hz) were collected from 51 stations at the different distance away from the epicenter of Kaikoura earthquake. Table 4 lists the station ID and the epicentral distance at each station. The location of the stations and the epicenter are shown in Figure 8.

We processed the GPS data during the time period from 11:02:45 to 11:07:45 in UTC, which includes the whole seismic period. The application to this seismic event further demonstrates the capability of retrieving coseismic displacement waveforms based on TPP method with real-time orbit and clock products. Similarly, the displacements were also calculated with TPP+CODE and VADASE+BRDC in this section. Furthermore, the postprocessing displacements were obtained by using CSRS-PPP online tool as references.

**Figure 9:**
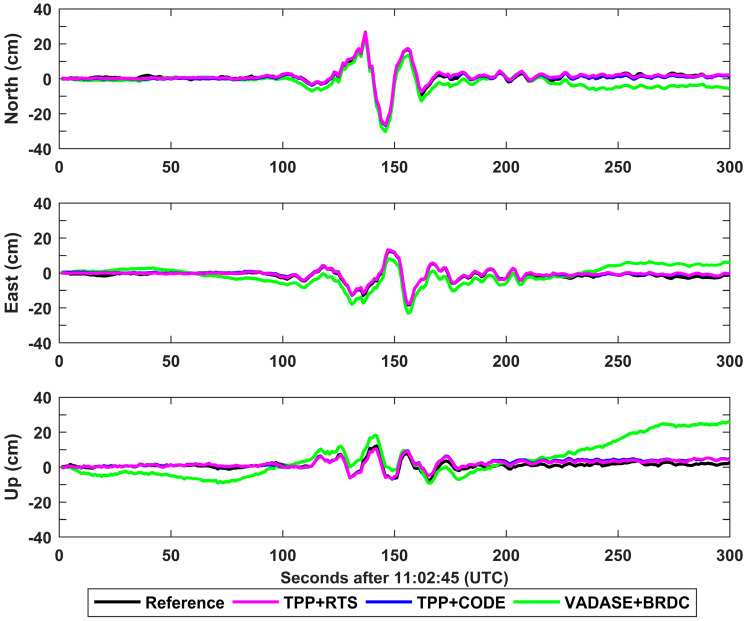
The retrieved coseismic displacements in north, east and up directions for different schemes at station WRPA.

Figure 9 shows the retrieved coseismic displacement waveforms at the station WRPA. WRPA is located in the southwestern of Masterton with an epicentral distance of about 280.13 km. Both the displacements derived from TPP+RTS and TPP+CODE fit with the references, obtained from the CSRS-PPP, very well. The average biases in north, east and up directions are 2.1 cm, 2.2 cm and 3.8 cm for TPP+RTS. As for TPP+CODE, the average biases in the three directions are 1.9 cm, 2.0 cm and 3.7 cm. However, obvious drifts are displayed in the displacements derived from VADASE+BRDC, the biases in the three directions reach up to 7.5 cm, 7.7 cm and 23.6 cm at the end of time series. 

**Figure 10:**
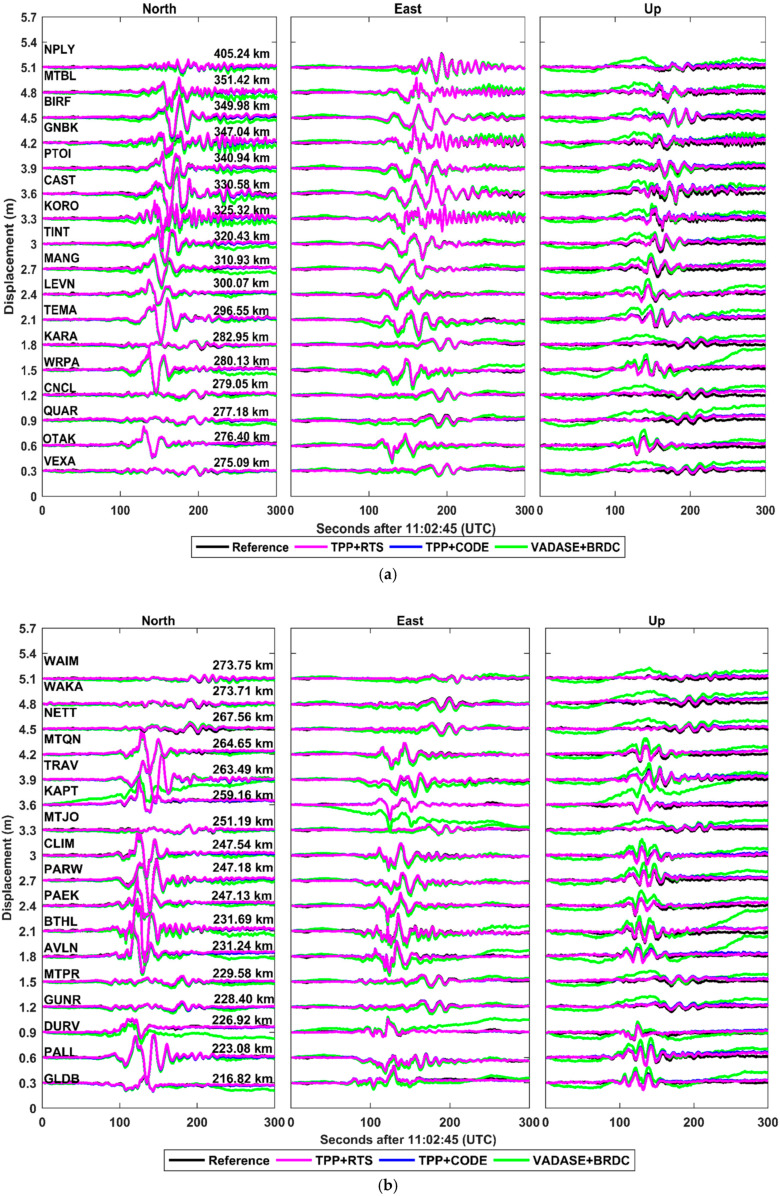

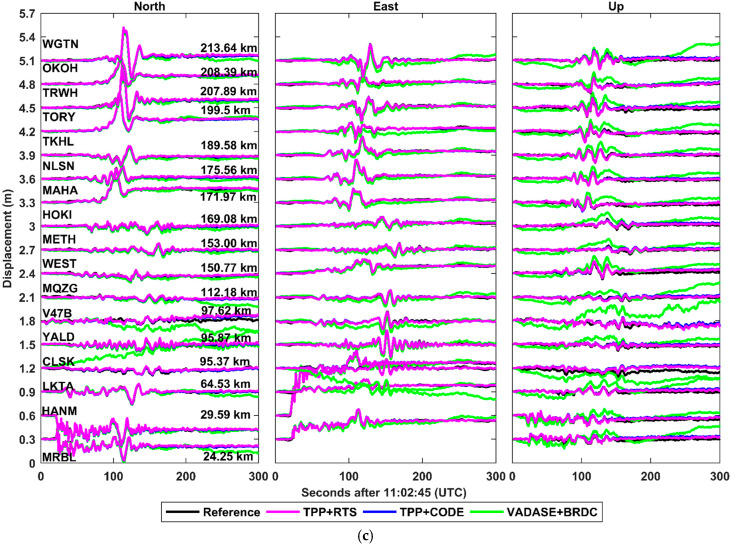
The coseismic displacement waveforms in north, east and up directions derived from different schemes at the selected 51 stations with the epicentral distances ranging from 270–400 km (**a**), 210–270 km (**b**) and 24–210 km (**c**).

To qualitatively describe the seismic rupture propagation, the coseismic displacement waveforms at 51 stations are presented in Figure 10. It should be noticed that the coseismic displacements of each station are vertically shifted according to the epicentral distance. As shown in Figure 10, during the Kaikōura earthquake, seismic wave first arrived at MRBL and sequentially propagated to farther stations. For TPP+RTS, the displacements of MRBL and HANM, the two closest stations to the epicenter, have the peak-to-peak amplitudes of 37 cm to 43 cm, 38 cm to 82 cm and 12 to 19 cm in north, east and up directions, respectively. Obvious permanent coseismic offsets in north and east directions are observed at the stations MRBL and HANM. In addition, two clear separate bursts of energy release are also significant in the displacement waveforms at these two stations. At the northeastern area of the epicenter, the stations with epicentral distance ranging from 200 km to 350 km have obvious seismic signals with the peak-to-peak amplitudes of 23 cm to 75 cm, 17 cm to 40 cm and 12 cm to 23 cm in the three directions. Nevertheless, at the same epicentral distance, faint signals are observed from the stations located at southwestern area of epicenter, ranging from 7 cm to 18 cm, 8 cm to 24 cm and 5 to 21 cm. The possible reason for the vibration amplification effect is that two displacement pulses almost overlapped at the northeastern area of the epicenter because the rupture front propagated along the north direction [59,60]. Very similar seismic signals can be observed from the displacements derived from TPP+CODE. Both the displacements derived from TPP+RTS and TPP+CODE in north, east and up directions are in good agreement with the references obtained from the CSRS-PPP. The displacements derived from VADASE+BRDC have relatively small drifts in north and east directions and seismic signals can be approximately discerned in these two directions. However, it is difficult to identify seismic signals in up direction due to the displacement drifts, which might cause a misjudgment of seismic rupture propagation in real-time condition. 

With the CSRS-PPP-derived displacements as references, the average RMS values of the displacement biases over selected 51 stations were calculated for three schemes and the results are presented in Table 5. The average RMS values of the displacement biases in horizontal and vertical directions are 1.2 cm and 2.4 cm for TPP+RTS, 1.1 cm and 2.4 cm for TPP+CODE. While the accuracy of VADASE+BRDC in horizontal and vertical directions are 4.6 cm and 7.2 cm, respectively. In a word, there is almost no difference between the accuracies of coseismic displacements derived from TPP+RTS and TPP+CODE, and both these two schemes can provide much more precise coseismic displacement than the VADASE method. 

## 4. Conclusions

This contribution evaluates the performance of the real-time coseismic displacement retrieval based on TPP method with real-time orbit and clock products. The real-time precise orbit and clock offsets were recovered from RTS correction products. The temporal-differenced IF combinations were formed and adopted as TPP measurements. By applying this orbit and clock offsets, the coseismic displacement can be real-timely retrieved based on TPP measurements. The whole procedure of real-time displacement retrieval with TPP method based on IGS RTS correction products was presented in this contribution. Stationary experiment and an application to the 2016 Mw 7.8 Kaikōura earthquake were carried out to assess the accuracy of displacement derived from TPP method based on real-time orbit and clock products. The TPP method based on the CODE final products and VADASE method based on broadcast ephemeris were also implemented in these two experiments for comparison. In general, the accuracies of the displacements derived from TPP method with real-time orbit and clock products and CODE final products are nearly at the same level. There is almost no drift in displacement derived from the TPP method with real-time orbit and clock products compared to VADASE-retrieved displacement. In the stationary experiment, the displacement derived from TPP method with real-time orbit and clock products are at an accuracy of 1.8 cm in horizontal direction and 4.1 cm in vertical direction during the time period of 15 min. In the second experiment, the TPP method based on real-time orbit and clock products can provide coseismic displacement waveform at the accuracy of 1.2 cm and 2.4 cm in the horizontal and vertical directions with the postprocessing displacement derived from CSRS-PPP online tool as references. The contribution shows that IGS RTS corrections provide an open-access way for users to carry out real-time coseismic displacement retrieval. With the growing availability and reliability of the real-time orbit and clock products, TPP method based on IGS RTS corrections is gradually becoming a powerful tool to support the rapid hazard assessment and earthquake early warning.

## Figures and Tables

**Table 1:** Three processing schemes.

Scheme	Method	Orbit/Clock	Latency
TPP+RTS	TPP method	RTS	Real time
TPP+CODE	TPP method	CODE	Available after about two weeks
VADASE+BRDC	VADASE method	BRDC	Real time

**Table 2:** Data processing strategies for TPP method.

Items	Processing Information
Observations	GPS L1/L2
Elevation mask	10 degrees
Observation weight	Elevation-dependent weight; 3 mm for GPS raw carrier-phase
Antenna phase center	Both PCO and PCV at satellite and receiver were corrected with IGS antenna file [51]
Sagnac effect	Corrected by empirical model [52]
Special relativistic effect	Corrected by empirical model [52]
Shapiro time delay	Corrected by empirical model [52]
Phase windup	Corrected by empirical model [53]
Solid tide	Corrected according to IERS ^1^ Convention 2010 [54]
Ocean loading	Corrected according to IERS Convention 2010 [54]
Pole tide	Corrected according to IERS Convention 2010 [54]

^1^ IERS, International Earth Rotation and Reference Systems Service.

**Table 3:** The average RMS values of the retrieved displacements over 33 IGS stations for three different schemes.

Scheme	Horizontal (cm)	Vertical (cm)
TPP+RTS	1.8	4.1
TPP+CODE	1.7	3.8
VADASE+BRDC	12.1	15.7

**Table 4:** The epicentral distances of the selected GPS stations.

ID	Distance (km)	ID	Distance (km)	ID	Distance (km)
MRBL	24.25	GLDB	216.82	VEXA	275.09
HANM	29.59	PALI	223.08	OTAK	276.40
LKTA	64.53	DURV	226.92	QUAR	277.18
CLSK	95.37	GUNR	228.4	CNCL	279.05
YALD	95.87	MTPR	229.58	WRPA	280.13
V47B	97.62	AVLN	231.24	KARA	282.95
MQZG	112.18	BTHL	231.69	TEMA	296.55
WEST	150.77	PAEK	247.13	LEVN	300.07
METH	153.00	PARW	247.18	MANG	310.93
HOKI	169.08	CLIM	247.54	TINT	320.43
MAHA	171.97	MTJO	251.19	KORO	325.32
NLSN	175.56	KAPT	259.16	CAST	330.58
TKHL	189.58	TRAV	263.49	PTOI	340.94
TORY	199.5	MTQN	264.65	GNBK	347.04
TRWH	207.89	NETT	267.56	BIRF	349.98
OKOH	208.39	WAKA	273.71	MTBL	351.42
WGTN	213.64	WAIM	273.75	NPLY	405.24

**Table 5:** The average RMS values of coseismic displacement biases over selected 51 stations for three schemes.

Scheme	Horizontal (cm)	Vertical (cm)
TPP+RTS	1.2	2.4
TPP+CODE	1.1	2.4
VADASE+BRDC	4.6	7.2

## Data Availability

High-rate RINEX observation files for stationary experiment and New Zealand earthquake can be downloaded at https://cddis.nasa.gov/ and ftp://ftp.geonet.org.nz/. The CODE final orbit and clock products are available at ftp://igs.gnsswhu.cn/.
